# Clinicopathological features and survival outcomes of HPV-independent versus HPV-associated cervical adenocarcinoma: a Systematic Review and meta-analysis

**DOI:** 10.3389/fonc.2026.1837655

**Published:** 2026-05-08

**Authors:** Dan Wu, Yun Li, Yuehua Lang, Xuan Hong, Jianping Kong

**Affiliations:** Department of Gynecology, The First People’s Hospital of Jiande, Jiande, China

**Keywords:** cervical adenocarcinoma, HPV-associated, HPV-independent, meta-analysis, survival

## Abstract

**Background:**

Human papillomavirus (HPV)-independent cervical adenocarcinoma is increasingly recognized as a biologically and clinically distinct entity from HPV-associated adenocarcinoma. We aimed to compare the clinicopathological features and survival outcomes of HPV-independent versus HPV-associated cervical adenocarcinoma.

**Methods:**

We performed a systematic review and meta-analysis following PRISMA 2020 guidelines. PubMed, Web of Science, Scopus, Embase, and the Cochrane Library were searched from inception to 01 March 2026. Comparative studies evaluating HPV-independent/non-HPV-associated and HPV-associated cervical adenocarcinoma were included. Primary outcomes were overall survival (OS), disease-free survival (DFS), and progression-free survival (PFS). Secondary outcomes included distant metastasis/distant recurrence, local recurrence, and lymphovascular space invasion/lymphovascular invasion (LVSI/LVI) positivity. Hazard ratios (HRs) and odds ratios (ORs) with 95% confidence intervals (CIs) were pooled using fixed- or random-effects models according to heterogeneity.

**Results:**

HPV-independent/non-HPV-associated adenocarcinoma was associated with poorer OS (pooled HR 3.37, 95% CI 2.12–5.36) and PFS (pooled HR 2.94, 95% CI 2.01–4.31) than HPV-associated adenocarcinoma. DFS was worse in the primary analysis (pooled HR 2.56, 95% CI 1.15–5.72), but became non-significant in sensitivity analysis after exclusion of one study requiring reciprocal transformation (HR 2.02, 95% CI 0.87–4.66). HPV-independent/non-HPV-associated adenocarcinoma also showed higher risks of distant metastasis/distant recurrence (pooled OR 2.76, 95% CI 1.88–4.05), local recurrence (pooled OR 2.76, 95% CI 1.82–4.19), and LVSI/LVI positivity (pooled OR 3.00, 95% CI 2.07–4.35).

**Conclusions:**

HPV-independent/non-HPV-associated cervical adenocarcinoma is associated with more aggressive clinicopathological features and worse survival outcomes than HPV-associated adenocarcinoma. These findings support the distinct clinical behavior of HPV-independent disease and may inform risk stratification and management.

**Systematic Review Registration:**

PROSPERO, identifier CRD420261348146.

## Introduction

Cervical cancer remains a major cause of cancer-related morbidity and mortality worldwide. According to GLOBOCAN 2022, there were 662,301 new cases and 348,874 deaths globally, underscoring the continuing burden of this disease despite advances in prophylactic vaccination and screening (1). Contemporary elimination strategies have therefore increasingly focused on HPV vaccination and primary HPV-based screening, reflecting the central etiologic role of high-risk HPV in cervical carcinogenesis (2).

Within this context, cervical adenocarcinoma presents diagnostic and clinical challenges that differ from those of squamous cell carcinoma. The heterogeneity of glandular lesions has become increasingly apparent with advances in gynecologic pathology. An important step forward was the introduction of the International Endocervical Adenocarcinoma Criteria and Classification (IECC), which reclassified endocervical adenocarcinomas according to HPV etiology rather than traditional morphologic features alone (3). This etiologic framework was subsequently incorporated into the 2020 WHO Classification of Female Genital Tumors, which recognizes HPV-associated and HPV-independent adenocarcinomas as biologically and clinically distinct entities (4).

This change in classification is more than a matter of terminology. The IECC system has shown better interobserver reproducibility than earlier morphology-based schemes and correlates closely with HPV status, supporting its value in routine diagnostic practice (5). More importantly, this distinction appears to have prognostic relevance (6). HPV-independent tumors include several aggressive histotypes, such as gastric-type, clear cell, mesonephric, and endometrioid adenocarcinomas (7). Emerging molecular evidence also suggests that these tumors differ from HPV-driven lesions, with recurrent alterations involving TP53 and the PI3K/AKT signaling pathway (8, 9).

Retrospective cohort studies have consistently linked HPV-independent adenocarcinoma to less favorable clinicopathological features and poorer oncologic outcomes. Compared with HPV-associated tumors, HPV-independent tumors have been associated with older age at presentation, larger tumor size, deeper stromal invasion, and higher rates of lymphovascular space invasion (LVSI), as well as increased recurrence and worse survival (6, 8, 10). Similar findings have been reported across different treatment settings, including surgery, radiotherapy, and the management of locally advanced disease, suggesting that the adverse behavior of HPV-independent tumors is not limited to a single therapeutic context (11–13).

However, the available evidence remains difficult to interpret as a whole. Most studies are retrospective, with differences in sample size, case definition, use of ancillary HPV-related testing, treatment background, and endpoint reporting. As a result, although current data suggest a more aggressive phenotype for HPV-independent disease, the magnitude and consistency of these differences have not yet been clearly established through pooled comparative evidence (7).

To address this gap, we conducted a systematic review and meta-analysis to compare the clinicopathological characteristics and survival outcomes of HPV-independent versus HPV-associated cervical adenocarcinoma. Our aim was to determine whether HPV-independent disease is consistently associated with adverse pathological features and poorer prognosis, and to provide clearer evidence to support risk stratification and clinical management.

## Methods

### Protocol and reporting standard

This systematic review and meta-analysis was conducted in accordance with the Preferred Reporting Items for Systematic Reviews and Meta-Analyses (PRISMA) 2020 statement (14) and the methodological guidance provided in the Cochrane Handbook for Systematic Reviews of Interventions (15). The review protocol was prospectively registered in the International Prospective Register of Systematic Reviews (PROSPERO; registration number: CRD420261348146). The key methodological procedures, including literature search, study selection, data extraction, quality assessment, and quantitative synthesis, were defined in advance to enhance methodological rigor and transparency.

### Search strategy

A comprehensive literature search was independently performed by two reviewers in PubMed, Embase, Web of Science Core Collection, Scopus, and the Cochrane Library from database inception to 01 March 2026. The search strategy combined controlled vocabulary and free-text terms related to cervical/endocervical adenocarcinoma, HPV-independent/non-HPV-associated and HPV-associated classification, and clinicopathological or survival outcomes. Search terms were adapted to the syntax and indexing system of each database. In addition, the reference lists of all eligible studies and relevant review articles were manually screened to identify any additional studies. The full electronic search strategies for all databases are provided in the **Supplementary Material**.

### Inclusion and exclusion criteria

Studies were considered eligible if they met the following criteria: (1) the study population consisted of patients with histologically confirmed primary cervical or endocervical adenocarcinoma; (2) the study compared HPV-independent/non-HPV-associated adenocarcinoma with HPV-associated adenocarcinoma, based on IECC/WHO criteria, HPV testing, p16 immunohistochemistry, or other clearly defined histopathological classification systems; and (3) at least one outcome of interest, including survival outcomes or clinicopathological variables, was reported in a manner that allowed comparative extraction.

The primary outcomes were overall survival (OS), disease-free survival (DFS), and progression-free survival (PFS). Secondary outcomes included distant metastasis/distant recurrence, local recurrence, and lymphovascular space invasion/lymphovascular invasion (LVSI/LVI) positivity.

Studies were excluded if they met any of the following criteria: (1) non-comparative studies without extractable data for both HPV-independent and HPV-associated groups; (2) studies involving non-adenocarcinoma cervical histologies or mixed histological populations without separate adenocarcinoma data; (3) reviews, editorials, letters, conference abstracts, case reports, or other non-original articles; (4) duplicate or overlapping publications, in which case the most informative or methodologically appropriate dataset was retained for the primary analysis; or (5) studies with insufficient or unusable data for quantitative synthesis.

### Study selection

Study selection was carried out independently by two reviewers in two stages. After removal of duplicate records, titles and abstracts were screened for potential relevance. Full-text articles of potentially eligible studies were then assessed against the predefined inclusion and exclusion criteria. Any discrepancies were resolved through discussion, and a third reviewer was consulted when consensus could not be reached.

### Data extraction

Data were independently extracted by two reviewers using a standardized data collection form. The following information was collected from each eligible study: first author, publication year, country, study design, study population, total sample size, number of HPV-independent/non-HPV-associated cases, number of HPV-associated cases, classification method, treatment setting, and reported clinicopathological variables. For quantitative synthesis, effect estimates including hazard ratios (HRs), odds ratios (ORs), and their corresponding 95% confidence intervals (CIs) were extracted.

When HRs or CIs were not directly reported, they were derived from the available published data or figures whenever feasible. For studies in which the reported comparison direction differed from the prespecified analytic direction, reciprocal transformation was applied to ensure consistency across studies. When multiple publications appeared to involve overlapping cohorts, only one dataset was included in the primary analysis, while alternative datasets were considered in sensitivity analyses where appropriate.

### Quality assessment

The methodological quality of included non-randomized studies was independently evaluated by two reviewers using the Newcastle–Ottawa Scale (NOS), which assesses study quality across the domains of selection, comparability, and outcome/exposure, with a maximum score of 9 points. In line with common practice, studies with NOS scores of 5 or higher were considered to be of moderate-to-high quality. Any disagreements in quality assessment were resolved through discussion with a third reviewer.

### Statistical analysis

Meta-analyses were performed using Review Manager (RevMan) version 5.4.1. HRs and ORs with corresponding 95% CIs were pooled for time-to-event and dichotomous outcomes, respectively. For survival outcomes, HRs were converted to log(HR) values and standard errors before pooling. Statistical heterogeneity was assessed using Cochran’s Q test and the I² statistic, with P < 0.10 for the Q test and/or I² > 50% considered indicative of substantial heterogeneity. A fixed-effects model was used when heterogeneity was low, whereas a random-effects model was applied when substantial heterogeneity was present.

Sensitivity analyses were prespecified for outcomes in which additional assumptions were required during effect extraction or harmonization, including reciprocal transformation of reported comparisons, extraction of HRs from published figures, use of available-case denominators, or potential overlap between study cohorts. Formal assessment of publication bias was not performed because fewer than 10 studies were available for each pooled outcome. A two-sided P < 0.05 was considered statistically significant.

## Results

### Study selection and characteristics

**Figure 1:**
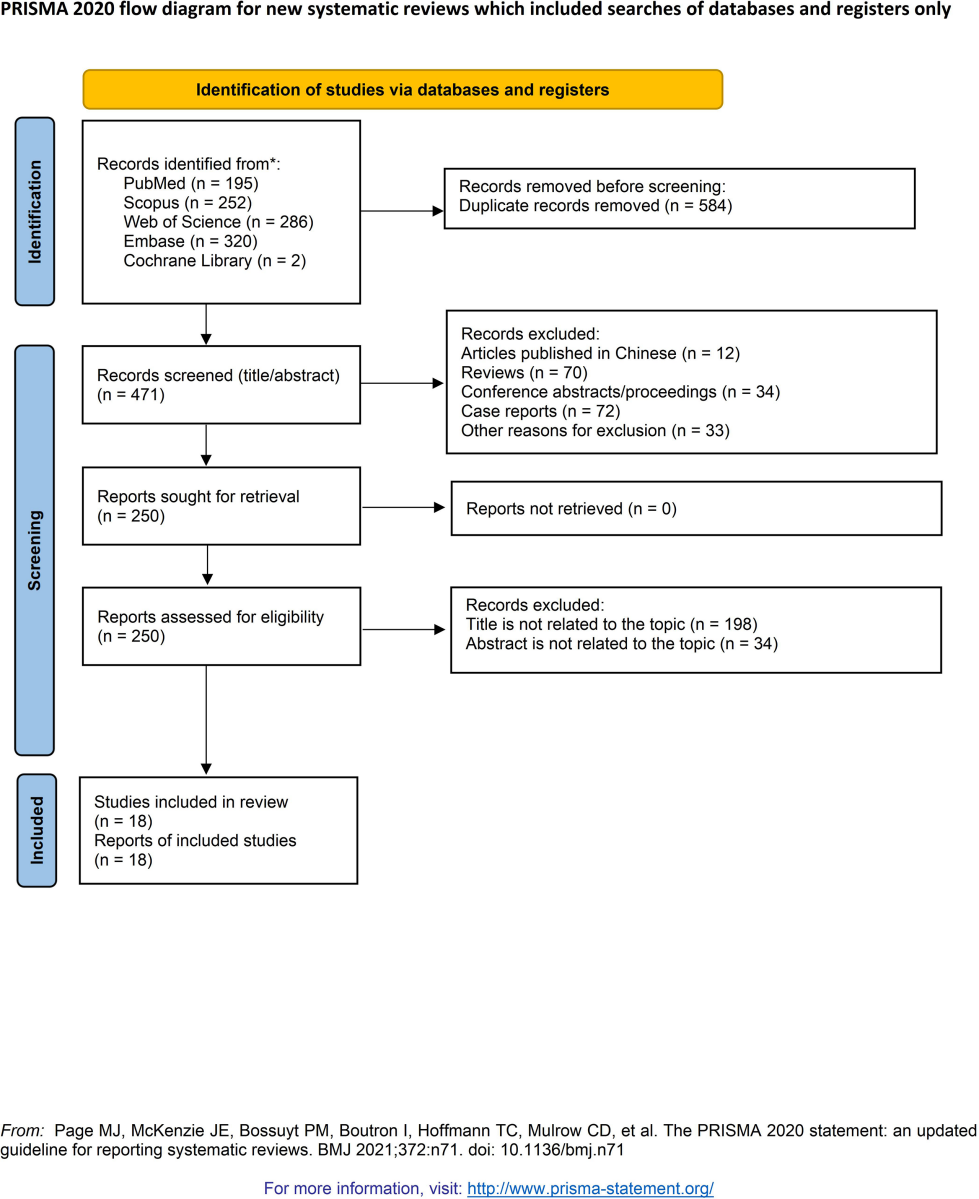
The PRISMA flow diagram displays the details of the selection process. *From: Page, M.J., McKenzie, J.E., Bossuyt, P.M., Boutron, I., Hofmann, T.C., Mulrow, C.D., Shamseer, L., Tetzlaf, J.M., Akl, E.A., Brennan, S.E., et al. (2021). The PRISMA 2020 statement: an updated guideline for reporting systematic reviews. Syst Rev. Mar 29;10(1):89. doi: 10.1186/s13643-021-01626-4. PMID: 33781348; PMCID: PMC8008539.

From database inception to 01 March 2026, the literature search identified 1055 records. After duplicate removal, title and abstract screening, and full-text review, 18 studies met the inclusion criteria and were included in the qualitative synthesis (3, 6, 8, 10–13, 16–26) (**Figure 1**); 12 of these contributed data to at least one quantitative analysis (6, 8, 10–13, 16, 19–23). All included studies were observational, and most were retrospective cohorts comparing clinicopathological features and survival outcomes between HPV-independent and HPV-associated cervical adenocarcinoma.

The studies varied considerably in classification criteria, HPV testing methods, treatment settings, and reported outcomes. While some cohorts consisted mainly of surgically treated patients (11, 21, 23), others included patients receiving radiotherapy or chemoradiotherapy (12, 13, 16), or mixed populations (3, 6, 8, 10, 17–20, 22, 24–26). As a result, the number of studies and patients contributing to each meta-analysis differed by outcome. In addition, inconsistent reporting required several prespecified sensitivity analyses, including reciprocal transformation of reported comparisons, extraction of hazard ratios from figures, and assessment of possible cohort overlap. Key study characteristics are summarized in **Table 1**. Detailed outcome data are provided in **Supplementary Table 1**, and quality assessment using the Newcastle–Ottawa Scale is shown in **Table 2**.

**Table 1:** Main characteristics of the studies included in the systematic review and meta-analysis.

Authors	Year	Country	Study design	Population/disease entity	Total (n)	HPVI (n)	HPVA (n)	Classification method	Treatment setting	Main clinicopathological variables reported	Survival outcomes
Baek et al. (16)	2025	Korea	Retrospective cohort	Endocervical adenocarcinoma, FIGO 2018 stage I–IVA, treated with definitive CCRT	40	18	22	Pathological review using IECC and 2020 WHO morphology-based classification into HPVA vs HPVI	Definitive CCRT	Stage; tumor size; tumor volume; parametrial invasion; LN metastasis; regressed tumor volume during RT; brachytherapy; OTT; recurrence pattern	CR; PFS; LRRFS; DMFS; OS
Ben-Mussa et al. (17)	2025	Northern Ireland, United Kingdom	Population-based retrospective cohort	Primary cervical adenocarcinoma	146	16	130	Specialist/central pathology review using current WHO HPV-associated vs HPV-independent classification; p16 performed	NR/treatment not uniformly reported	Age; stage at presentation; histologic subtype; mortality	Mortality/survival difference; median survival among deceased
Carvalho et al. (10)	2022	Brazil	Retrospective longitudinal single-center cohort	Endocervical adenocarcinoma morphologically reclassified by 2020 WHO criteria	117*	17	100	2020 WHO/IECC morphology-based reclassification into HPVA vs HPVI; p16 used only in equivocal cases	Mixed; treatment not significantly different between groups	Age; FIGO stage; relapse; death; BMI; mitotic index	DFS; OS
Cho et al. (11)	2022	Korea	Retrospective single-center cohort	Endocervical adenocarcinoma treated with radical hysterectomy and adjuvant RT	123	42	81	Single-pathologist morphology-based review using IECC criteria and updated WHO classification	Radical hysterectomy and pelvic LN dissection followed by adjuvant RT with or without concurrent chemotherapy	Age; tumor size; clinical/pathological FIGO stage; depth of invasion; parametrial invasion; vaginal resection margin; LVSI; LN involvement; adjuvant treatment; failure patterns	DFS; LRFS; RRFS; DMFS
Cho et al. (12)	2023	Korea	Retrospective multi-institutional cohort	Endocervical adenocarcinoma after radical hysterectomy	365	90	275	Morphology-based classification using 2020 WHO and IECC criteria; ancillary IHC/genetic data only for very few ambiguous cases	Radical hysterectomy and pelvic LN dissection with mixed adjuvant treatment (none/RT alone/CCRT/sequential CTx-RT/CTx alone)	Age; tumor size; FIGO stage; invasion depth; parametrial invasion; resection margin involvement; LVSI; para-aortic LN metastasis; adjuvant treatment; recurrence patterns	DFS; LRRFS; DMFS; OS
He et al. (18)	2025	China	Retrospective single-center cohort	Primary cervical adenocarcinoma	332	47	285	2020 WHO/IECC-based classification into HPV-Ind-CA vs HPV-CA	Mixed (surgery/adjuvant radiotherapy/adjuvant chemotherapy)	Age; menopausal status; contact bleeding; HPV infection status; CA125/CA199/CEA; surgery; adjuvant RT/chemotherapy; tumor size; differentiation; FIGO stage; pathologic subtype	PFS; OS
Hodgson et al. (6)	2019	Canada	Retrospective single-center cohort	Invasive endocervical adenocarcinoma	87	16	71	Morphology-based IECC classification by three gynecologic pathologists with majority/consensus assignment	Mixed (surgical resection or primary chemoradiation)	Age; horizontal extent; depth of invasion; LVSI; lymph node involvement; FIGO stage; Silva pattern; primary treatment modality	DFS; DSS
Matsubara et al. (19)	2025	Japan	Multicenter retrospective cohort	Pure invasive cervical adenocarcinoma, surgically treated, preoperative FIGO 2008 stage I–II, no gross residual disease	261*	60	201	Central morphology-based classification into HPVA vs HPVI according to the international group criteria and WHO 5th edition; HPVA further subclassified by Silva and binary Silva classification	Surgically treated cohort with mixed adjuvant therapy (none/adjuvant chemotherapy/adjuvant RT/adjuvant CCRT)	Age; MRI tumor size; preoperative stage; cervical stromal invasion; surgery type; pelvic/para-aortic node assessment; LVSI; lymph node metastasis; adjuvant therapy; recurrence pattern; death	DFS; OS
Ren et al. (8)	2021	Canada	Retrospective single-center resection cohort	Invasive endocervical adenocarcinoma	100	15	85	IECC classification using histology, p16 immunohistochemistry, and HPV RNA *in situ* hybridization	Hysterectomy/resection cohort with variable postsurgical adjuvant treatment	Age; tumor size; margin status; Silva pattern; grade; LVI; FIGO stage; postsurgical treatment; mutational findings	OS; DSS; PFS
Seki et al. (13)	2023	Japan	Multicenter retrospective cohort	Locally advanced cervical adenocarcinoma (FIGO 2018 IB3–IIIC1)	151	48	103	Central pathological review according to 2020 WHO morphology-based HPVi/HPVa classification, supported by p16/p53 immunohistochemistry; no HPV molecular testing	Locally advanced cohort treated regardless of primary modality; surgery predominant, RT/CCRT minority	Age category; FIGO stage; lymphadenopathy; parametrial invasion; tumor diameter; treatment method; neoadjuvant chemotherapy; adjuvant therapy	PFS; OS
Shi et al. (20)	2022	China	Retrospective single-center resection cohort	Resected endocervical adenocarcinoma	386*	88	298	WHO 2020/IECC morphology-based classification with ancillary p16 IHC and HPV RNA ISH	Resected surgical cohort	Age; tumor size; depth of invasion; upper genital tract spread; LVSI; nerve involvement; FIGO stage; p53 expression; precursor lesions; histologic subtype	OS; tumor recurrence
Stolnicu et al. (21)	2018	International	Multicenter retrospective cohort	Invasive endocervical adenocarcinoma	361*	55	306	IECC morphology-based classification, validated with p16 IHC and RNA-based high-risk HPV ISH	Surgical/excision cohort; no neoadjuvant therapy	Age; tumor size; FIGO stage; histologic subtype; precursor lesions; p16/HPV status	No dedicated survival comparison reported
Stolnicu et al. (22)	2019	International	Multicenter retrospective cohort	Endocervical adenocarcinoma	341	49	292	IECC morphology-based classification confirmed by high-risk HPV RNA ISH	Surgical/excision cohort; no neoadjuvant therapy	Tumor size; FIGO stage; LVI; lymph node metastasis; histologic subtype; Silva pattern	Recurrence and mortality reported for Silva pattern C subset
Stolnicu et al. (23)	2021	International	Multicenter retrospective cohort	Endocervical adenocarcinoma	205	36	169	IECC morphology-based classification with high-risk HPV RNA ISH confirmation	Surgical/excision cohort; surgery alone vs surgery + adjuvant treatment; no neoadjuvant therapy	Age; tumor size; FIGO stage; LVI; LNM; local recurrence; distant recurrence; histologic subtype	OS; DFS; PFS
Stolnicu et al. (3)	2018	International	International multicenter retrospective cohort	Endocervical adenocarcinoma	381*	52	329	IECC morphology-based classification within a FIGO 2018 stage IB cohort	Surgically treated stage IB cohort (conization/trachelectomy/hysterectomy), no neoadjuvant therapy	Age; surgical treatment; precursor lesions; HPV status; Silva pattern; tumor grade; LVSI; lymph node metastasis; recurrence; local recurrence; distant recurrence	RFS; OS
Williams et al. (24)	2026	USA	Retrospective cohort	Invasive endocervical adenocarcinoma	144	34	110	Integrated IECC-based classification combining morphology with available clinical digene HPV results and ancillary immunohistochemistry when available	Mixed primary treatment modality within a large integrated healthcare system (surgery alone/chemoradiation/multimodal)	Age; race/ethnicity; parity; BMI; histologic subtype; FIGO stage; CCI; NDI; treatment category; bevacizumab; immunotherapy; positive digene HPV testing	PFS; OS
Yasutake et al. (25)	2024	Japan	Retrospective cohort	Invasive endocervical adenocarcinoma	103*	25	78	WHO 5th morphology-based classification with p16 IHC, Rb IHC, and HR-HPV mRNA ISH	Mixed biopsy/resection cohort; patients subsequently treated with surgery or radiation	Clinicopathological features; p16 overexpression; Rb loss; HR-HPV association	OS
Zhang et al. (26)	2023	China	Retrospective multicenter cohort	Invasive endocervical adenocarcinoma	444*	72	372	WHO 2020 histologic classification with HPV DNA detection by WTS-PCR and selected LCM-PCR, plus p16/PR immunohistochemistry review	Pathology cohort (surgical resection and punch biopsy specimens); treatment not analyzed	Age; histologic subtype distribution; HPV positivity; HPV genotype distribution; p16/PR positivity	NR

BMI, body mass index; CA125, carbohydrate antigen 125; CA199, carbohydrate antigen 19-9; CCI, Charlson comorbidity index; CCRT, concurrent chemoradiotherapy; CI, confidence interval; CR, complete response; CT, chemotherapy; CTx, chemotherapy; DFS, disease-free survival; DMFS, distant metastasis-free survival; DNA, deoxyribonucleic acid; DSS, disease-specific survival; ECA, endocervical adenocarcinoma; FIGO, International Federation of Gynecology and Obstetrics; HPV, human papillomavirus; HPVA, HPV-associated adenocarcinoma; HPVI, HPV-independent adenocarcinoma; HR, hazard ratio; IHC, immunohistochemistry; IECC, International Endocervical Adenocarcinoma Criteria and Classification; ISH, *in situ* hybridization; ISMC, invasive stratified mucin-producing carcinoma; iSMILE, invasive stratified mucin-producing intraepithelial lesion; LCM-PCR, laser capture microdissection polymerase chain reaction; LNM, lymph node metastasis; LRFS, local recurrence-free survival; LRRFS, locoregional recurrence-free survival; LVI, lymphovascular invasion; LVSI, lymphovascular space invasion; MRI, magnetic resonance imaging; mRNA, messenger ribonucleic acid; NAC, neoadjuvant chemotherapy; NDI, neighborhood deprivation index; NHPVA, non-HPV-associated adenocarcinoma; NOS, not otherwise specified; NR, not reported; OS, overall survival; PFS, progression-free survival; PR, progesterone receptor; Rb, retinoblastoma protein; RFS, recurrence-free survival; RNA, ribonucleic acid; RRFS, regional recurrence-free survival; RT, radiotherapy; SD, stable disease; WHO, World Health Organization; WTS-PCR, whole tissue section polymerase chain reaction. * For selected studies, the total number shown in **Table 1** reflects the directly comparable analytic cohort for HPV-independent versus HPV-associated adenocarcinoma. **Supplementary Table 1** preserves the full reclassified cohort, including unclassified, NOS, or other cases when reported.

**Table 2:** The Newcastle-Ottawa scale (NOS) for assessing the quality of nonrandomized studies in our study.

Study	Year	Country	Type of article	The Newcastle-Ottawa Scale (NOS)		
				Selection	Comparability	Outcome
Baek et al. (16)	2025	Korea	Retrospective cohort	* * * *	* *	* * *
Ben-Mussa et al. (17)	2025	Northern Ireland, United Kingdom	Population-based retrospective cohort	* * * *	–	* * *
Carvalho et al. (10)	2022	Brazil	Retrospective longitudinal study	* * * *	* *	* * *
Cho et al. (11)	2022	Korea	Retrospective cohort	* * * *	* *	* * *
Cho et al. (12)	2023	Korea	Retrospective multi-institutional cohort	* * * *	* *	* * *
He et al. (18)	2025	China	Retrospective single-center cohort	* * * *	*	* *
Hodgson et al. (6)	2019	Canada	Retrospective single-center cohort	* * * *	*	* * *
Matsubara et al. (19)	2025	Japan	Multicenter retrospective cohort	* * * *	* *	* * *
Ren et al. (8)	2021	Canada	Retrospective single-center resection cohort	* * * *	* *	* * *
Seki et al. (13)	2023	Japan	Multicenter retrospective cohort	* * * *	* *	* * *
Shi et al. (20)	2022	China	Retrospective single-center resection cohort	* * * *	* *	* * *
Stolnicu et al. (21)	2018	International	Multicenter retrospective cohort	* * * *	–	*
Stolnicu et al. (22)	2019	International	Multicenter retrospective cohort	* * * *	*	* *
Stolnicu et al. (23)	2021	International	Multicenter retrospective cohort	* * * *	* *	* * *
Stolnicu et al. (3)	2018	International	International multicenter retrospective cohort	* * * *	* *	* * *
Williams et al. (24)	2026	USA	Retrospective cohort	* * * *	–	* * *
Yasutake et al. (25)	2024	Japan	Retrospective cohort	* * * *	* *	* *
Zhang et al. (26)	2023	China	Retrospective multicenter cohort	* * * *	–	*

* = 1 point; maximum score = 9 points.

NOS was applied using the cohort-study domains: Selection, Comparability, and Outcome.

“– “ indicates that the comparability domain was not applicable because the study was single-arm or lacked an external comparison cohort, and therefore could not be assessed according to the cohort Newcastle–Ottawa Scale.

### Primary outcomes

#### Overall survival

**Figure 2:**
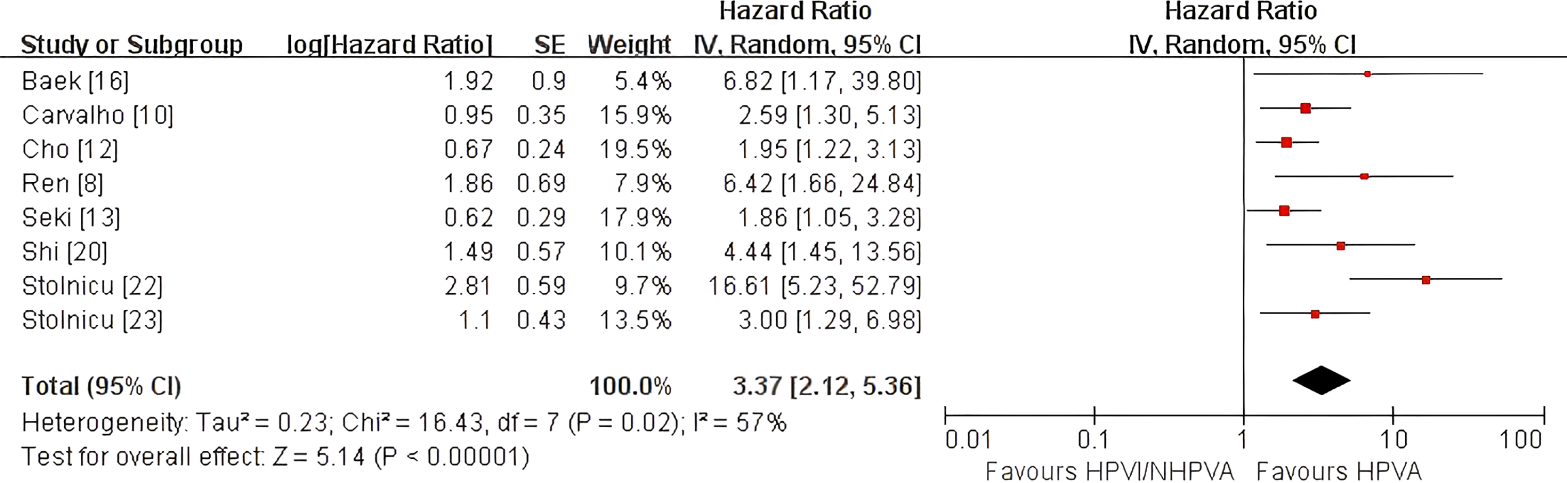
Random-effects meta-analysis of overall survival (OS) comparing HPV-independent with HPV-associated cervical adenocarcinoma, shown as a forest plot. OS, overall survival; HR, hazard ratio; CI, confidence interval.

Eight studies comprising 1,705 patients (8, 10, 12, 13, 16, 20, 22, 23) contributed to the meta-analysis of overall survival (OS). HPV-independent adenocarcinoma was associated with significantly poorer OS than HPV-associated disease (HR 3.37, 95% CI 2.12–5.36; P < 0.00001; **Figure 2**), with moderate heterogeneity (I² = 57%). Sensitivity analysis excluding three studies (8, 16, 22) that required additional assumptions for effect extraction or harmonization showed that the association remained statistically significant (HR 2.25, 95% CI 1.69–3.01; P < 0.00001; **Supplementary Figure 1**), while heterogeneity was no longer evident (I² = 0%). Re-inclusion of these studies increased both the magnitude of the pooled estimate and between-study heterogeneity, but the direction and statistical significance of the association remained unchanged. These findings support the robustness of the OS result.

#### Disease-free survival

**Figure 3:**
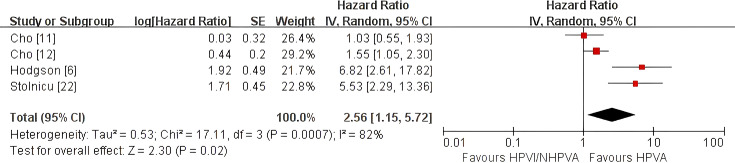
Random-effects meta-analysis of disease-free survival (DFS) comparing HPV-independent with HPV-associated cervical adenocarcinoma, shown as a forest plot. DFS, disease-free survival; HR, hazard ratio; CI, confidence interval.

Four studies comprising 916 patients (6, 11, 12, 22) were included in the primary meta-analysis of disease-free survival (DFS). Random-effects pooling showed significantly poorer DFS in HPV-independent adenocarcinoma (HR 2.56, 95% CI 1.15–5.72; P = 0.02; **Figure 3**), although substantial heterogeneity was present (I² = 82%). When study (22), which required reciprocal transformation to align the reported comparison direction with the prespecified analytic framework, was excluded, the pooled estimate became non-significant (HR 2.02, 95% CI 0.87–4.66; P = 0.10; **Supplementary Figure 2**), while heterogeneity remained high (I² = 81%). Thus, although the direction of effect remained consistent, the statistical significance of the DFS result was sensitive to the inclusion of studies requiring additional data harmonization.

#### Progression-free survival

**Figure 4:**
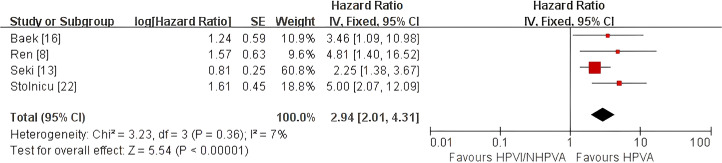
Fixed-effect meta-analysis of progression-free survival (PFS) comparing HPV-independent with HPV-associated cervical adenocarcinoma, shown as a forest plot. PFS, progression-free survival; HR, hazard ratio; CI, confidence interval.

Four studies comprising 632 patients (8, 13, 16, 22) were analyzed for progression-free survival (PFS). HPV-independent adenocarcinoma was associated with significantly poorer PFS than HPV-associated disease (HR 2.94, 95% CI 2.01–4.31; P < 0.00001; **Figure 4**), with low heterogeneity (I² = 7%). Sensitivity analysis excluding study (16), a chemoradiotherapy-only cohort with potential inconsistency in the reported effect direction, and study (8), which required figure-based HR extraction and reciprocal transformation, yielded a similarly significant result (HR 3.07, 95% CI 1.43–6.60; P = 0.004; **Supplementary Figure 3**). Although moderate heterogeneity was observed in this reduced dataset (I² = 59%), this should be interpreted cautiously because only two studies remained. Overall, the PFS result appeared stable.

### Secondary outcomes

#### Distant metastasis/distant recurrence

**Figure 5:**
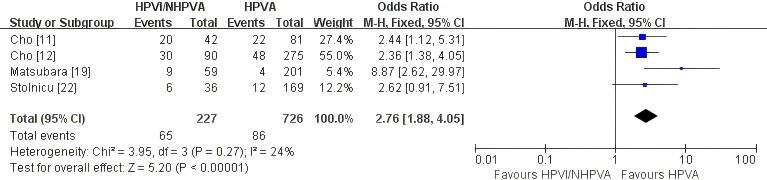
Fixed-effect meta-analysis of distant metastasis/distant recurrence comparing HPV-independent with HPV-associated cervical adenocarcinoma, shown as a forest plot. OR, odds ratio; CI, confidence interval.

Four studies comprising 953 patients (11, 12, 19, 22) were included in the meta-analysis of distant metastasis/distant recurrence. HPV-independent adenocarcinoma was associated with a significantly higher risk of distant events than HPV-associated disease (OR 2.76, 95% CI 1.88–4.05; P < 0.00001; **Figure 5**), with low heterogeneity (I² = 24%).

#### Local recurrence

**Figure 6:**
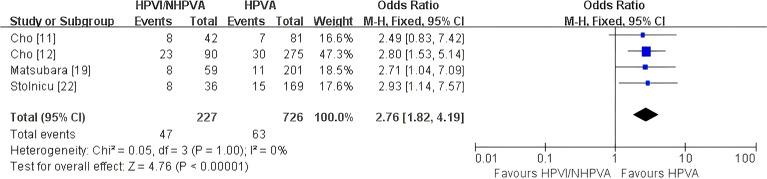
Fixed-effect meta-analysis of local recurrence comparing HPV-independent with HPV-associated cervical adenocarcinoma, shown as a forest plot. OR, odds ratio; CI, confidence interval.

Four studies comprising 953 patients (11, 12, 19, 22) contributed to the analysis of local recurrence. HPV-independent adenocarcinoma was associated with a significantly higher risk of local recurrence than HPV-associated disease (OR 2.76, 95% CI 1.82–4.19; P < 0.00001; **Figure 6**). No heterogeneity was observed across studies (I² = 0%).

#### LVSI/LVI positivity

**Figure 7:**
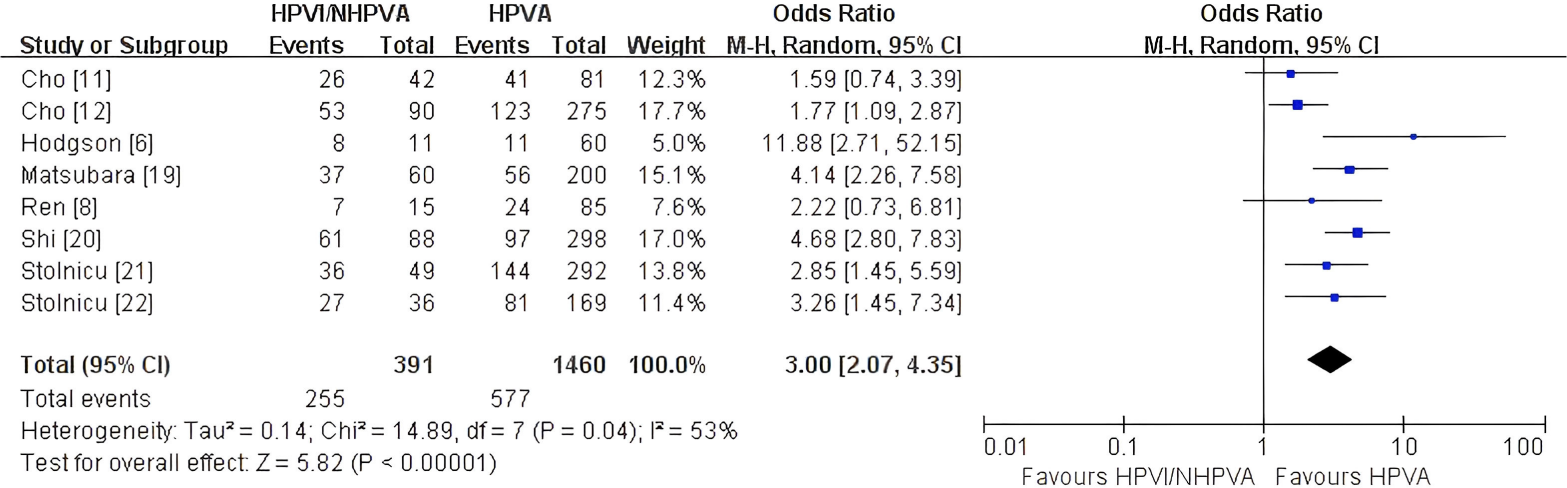
Random-effects meta-analysis of lymphovascular space invasion/lymphovascular invasion (LVSI/LVI) positivity comparing HPV-independent with HPV-associated cervical adenocarcinoma, shown as a forest plot. LVSI, lymphovascular space invasion; LVI, lymphovascular invasion; OR, odds ratio; CI, confidence interval.

Eight studies comprising 1,851 patients (6, 8, 11, 12, 19–22) evaluated lymphovascular space invasion (LVSI)/lymphovascular invasion (LVI) positivity. HPV-independent tumors were significantly more likely to show LVSI/LVI positivity than HPV-associated tumors (OR 3.00, 95% CI 2.07–4.35; P < 0.00001; **Figure 7**), with moderate heterogeneity (I² = 53%). Sensitivity analysis excluding study (6), which reported LVSI using available cases rather than the full cohort denominator, and study (22), which was considered potentially overlapping with the international cohort reported in study (21), showed that the association remained statistically significant and in the same direction (OR 2.74, 95% CI 1.84–4.07; P < 0.00001; **Supplementary Figure 4**), with persistent moderate heterogeneity (I² = 55%). These findings support the robustness of the association between HPV-independent status and LVSI/LVI positivity.

## Discussion

Our meta-analysis showed that HPV-independent cervical adenocarcinoma is associated with a more aggressive clinicopathological phenotype and poorer oncologic outcomes than HPV-associated disease. Pooled estimates demonstrated significantly worse overall survival and progression-free survival in HPV-independent tumors. These tumors were also associated with higher risks of distant metastasis, local recurrence, and lymphovascular space invasion. For disease-free survival, although the primary analysis suggested a significant disadvantage, this association lost statistical significance in sensitivity analysis after exclusion of the study by Stolnicu et al., which required reciprocal transformation of the reported comparison. This finding suggests that the evidence for DFS differences, while directionally consistent, should be interpreted with caution. Overall, these results support the view that HPV-independent adenocarcinoma is a biologically distinct subtype with more aggressive clinical behavior, rather than one that differs from HPV-associated adenocarcinoma only in morphology.

OS and PFS appeared to be the most stable survival endpoints in this study. For OS, the pooled estimate remained statistically significant after exclusion of studies requiring additional assumptions for effect harmonization, although the effect size was attenuated from 3.37 to 2.25 and heterogeneity was no longer evident. A similar pattern was observed for PFS, which remained significant after exclusion of studies with concerns regarding reporting direction or figure-derived extraction. These findings suggest that the adverse survival signal in HPV-independent disease is unlikely to be explained solely by a single influential study or by the methodological handling of a limited number of datasets. This interpretation is supported by prior cohort studies across different clinical settings. In the multi-institutional KROG 20–07 study, HPV-independent status was independently associated with worse OS (HR 2.48, 95% CI 1.59–3.88) and DFS (HR 1.92, 95% CI 1.32–2.78), together with higher rates of local recurrence (25.6% vs. 10.9%) and distant metastasis (33.3% vs. 17.5%) (12). Similarly, in a definitive chemoradiotherapy cohort, Baek et al. reported poorer 3-year PFS (16.7% vs. 49.8%) and OS (42.3% vs. 90.7%) for HPV-independent versus HPV-associated disease, with corresponding multivariable HRs of 3.44 and 6.83 (16). More recent multicontinental and treatment-modality-based studies further support the prognostic relevance of HPV status, while also suggesting that the magnitude of risk may vary according to stage distribution and treatment context (27, 28). However, formal comparison according to treatment modality was not feasible in the present meta-analysis because most included studies reported mixed treatment populations or did not provide stratified outcome data. Notably, the adverse prognostic pattern of HPV-independent disease appeared consistent across surgical, chemoradiotherapy, and mixed cohorts, supporting the interpretation that our pooled OS and PFS results reflect a genuinely aggressive clinical phenotype rather than methodological variation alone.

Unlike OS and PFS, the DFS result was more sensitive to study selection and analytic assumptions. In the primary analysis, HPV-independent adenocarcinoma was associated with significantly poorer DFS (HR 2.56), but this association lost statistical significance after exclusion of the study by Stolnicu et al. (22), which required reciprocal transformation to align the reported comparison with our prespecified analytic direction. Importantly, the pooled estimate remained directionally consistent (HR 2.02), although heterogeneity remained high. This instability likely reflects the small number of contributing studies and the lack of uniformity in recurrence-related endpoints across cohorts. Nevertheless, the direction of effect is supported by individual studies: Stolnicu et al. (23) reported poorer recurrence-free survival in HPV-independent stage IB tumors, with 5-year rates of 63% versus 84% in HPV-associated disease, and He et al. (18) observed shorter median progression-free survival in HPV-independent disease (12 vs. 26 months). Similar adverse recurrence patterns have also been reported in gastric-type adenocarcinoma, a representative HPV-independent subtype. Nishio et al. found significantly poorer DFS and OS in gastric-type mucinous carcinoma than in usual-type endocervical adenocarcinoma in a multi-institutional study (both P < 0.0001) (29), and Xi et al. reported higher recurrence/metastasis rates in gastric-type adenocarcinoma than in HPV-associated adenocarcinoma (30). Because differences in time origin, event definition, and censoring can materially affect cross-study comparisons (31), the DFS result in the present study should be interpreted as supportive rather than conclusive evidence of an adverse prognosis in HPV-independent disease.

The recurrence and LVSI findings provide important pathological context for the survival differences observed in our study. In our pooled analysis, HPV-independent tumors exhibited significantly higher odds of both distant metastasis and local recurrence (OR 2.76 for each), together with a threefold higher likelihood of LVSI positivity (OR 3.00). The association with LVSI also remained stable in sensitivity analysis (OR 2.74). These findings suggest that the adverse prognosis of HPV-independent disease is not reflected only in survival outcomes, but is also supported by a more invasive phenotype with a greater tendency for dissemination. This interpretation is consistent with the surgical cohort reported by Cho et al., who found lower local recurrence-free survival (77.3% vs. 91.8%) and distant metastasis-free survival (50.1% vs. 73.7%) in HPV-independent tumors, together with higher rates of positive vaginal margins and peritoneal seeding (11). Together, these findings provide pathological support for the more aggressive recurrence profile observed in HPV-independent adenocarcinoma.

This invasive behavior is particularly evident in gastric-type adenocarcinoma, the prototypical HPV-independent subtype. Contemporary studies have consistently reported poor outcomes in this subgroup: Ehmann et al. reported median PFS and OS of 17 and 33 months, respectively, in stage II–IV disease (32); Chang et al. found that more than 60% of patients presented with invasion or metastasis, with a 5-year OS rate of 46.1% (33); and larger series have described high nodal burden, frequent recurrence, and short survival (34). These findings support the biological plausibility of the poorer OS, PFS, and recurrence profile observed in HPV-independent adenocarcinoma.

Our findings support the clinical relevance of the dualistic etiologic classification adopted by the IECC and WHO. Rather than representing simply morphological variants of HPV-associated disease, HPV-independent adenocarcinomas constitute a pathogenetically distinct group characterized by unique biological drivers and more aggressive clinical behavior (3, 4). This group includes diverse histotypes, such as gastric-type, clear cell, mesonephric, and endometrioid adenocarcinomas, with gastric-type carcinoma being the most prevalent and clinically aggressive subtype (7, 35). The poorer OS, PFS, and recurrence profile observed in our meta-analysis is consistent with the adverse clinical course reported for these histotypes, particularly gastric-type tumors (13, 32–34).

This clinical behavior is further supported by a distinct molecular landscape. Unlike HPV-driven tumors, which depend largely on viral oncoproteins E6 and E7, HPV-independent cancers more often harbor somatic alterations involving TP53, PIK3CA, KRAS, STK11, and PTEN, with dysregulation of pathways such as PI3K/AKT (8, 9, 36). These non-viral genomic alterations may contribute to recurrence, dissemination, and potentially less favorable responses to conventional treatment. Therefore, the poor prognosis of HPV-independent adenocarcinoma is likely rooted in its underlying biology rather than representing an isolated statistical observation.

From a clinical perspective, accurate distinction between HPV-independent and HPV-associated adenocarcinoma is important for prognostic assessment, patient counseling, follow-up planning, and risk stratification within the IECC/WHO framework (3, 4, 35). Although current cervical cancer management remains predominantly stage-based and the available evidence does not yet justify subtype-specific therapeutic protocols, contemporary guidelines emphasize expert pathology review and recognition of aggressive histological subtypes in multidisciplinary decision-making (37). Previous cohort studies have suggested poorer prognosis for HPV-independent disease, but interpretation has been limited by heterogeneity in classification methods, sample sizes, treatment settings, and endpoints (6, 10–13, 16, 18, 22–24). By pooling comparative data and incorporating sensitivity analyses, our study provides a clearer quantitative estimate of these prognostic differences and helps consolidate evidence supporting the role of HPV status in pathological classification, prognostication, and risk stratification in cervical adenocarcinoma (38–40).

Our study has several strengths. First, it followed PRISMA 2020 guidelines, was prospectively registered, and used a rigorous methodology, including a comprehensive database search, independent dual review, standardized data extraction, and quality assessment. Second, we evaluated multiple clinically relevant endpoints, including OS, PFS, DFS, distant metastasis, local recurrence, and LVSI/LVI, thereby providing a broader assessment of the clinical and pathological behavior of HPV-independent adenocarcinoma. Third, we performed prespecified sensitivity analyses to address inconsistent reporting, reciprocal transformation of effect estimates, figure-derived HR extraction, available-case denominators, and possible cohort overlap. These analyses helped distinguish robust findings from those more sensitive to methodological assumptions.

This study also has several limitations. Most included studies were retrospective, introducing the possibility of selection bias and residual confounding that could not be fully addressed. There was also substantial heterogeneity across studies in classification criteria, HPV testing methods, treatment approaches, and follow-up duration. For some outcomes, particularly disease-free and progression-free survival, the small number of available studies limited statistical power and precluded formal assessment of publication bias. The need for multiple sensitivity analyses, driven by inconsistent reporting formats and possible cohort overlap, also reflects the limited standardization of the current literature. Finally, incomplete reporting of key prognostic factors, including stage distribution, treatment intensity, and specific molecular alterations, prevented more detailed subgroup analyses that might have helped explain sources of heterogeneity.

In conclusion, HPV-independent cervical adenocarcinoma is associated with poorer survival outcomes, higher recurrence risk, and more aggressive pathological features than HPV-associated disease. These findings support its recognition as a distinct high-risk subtype and highlight the importance of accurate etiologic classification for risk stratification, follow-up planning, and clinical decision-making. However, because the available evidence is mainly retrospective and heterogeneous, these results should be interpreted with caution. Future prospective studies using standardized diagnostic criteria and uniform endpoint definitions are needed to clarify subtype-specific prognostic risk and management strategies.

## Data Availability

The data analyzed in this study is subject to the following licenses/restrictions: Any researchers interested in this study could contact Dan Wu (E-mail: wd15757153153@163.com) to request the data. Requests to access these datasets should be directed to wd15757153153@163.com.

## Supplementary material

Supplementary Figure 1 Sensitivity analysis of overall survival (OS) after exclusion of studies requiring additional assumptions for effect extraction or harmonization, shown as a fixed-effect forest plot. OS, overall survival; HR, hazard ratio; CI, confidence interval.

Supplementary Figure 2 Sensitivity analysis of disease-free survival (DFS) after exclusion of the study requiring reciprocal transformation to align the reported comparison with the prespecified analytic direction, shown as a random-effects forest plot. DFS, disease-free survival; HR, hazard ratio; CI, confidence interval.

Supplementary Figure 3 Sensitivity analysis of progression-free survival (PFS) after exclusion of studies with concerns regarding reporting direction or figure-derived extraction, shown as a random-effects forest plot. PFS, progression-free survival; HR, hazard ratio; CI, confidence interval.

Supplementary Figure 4 Sensitivity analysis of lymphovascular space invasion/lymphovascular invasion (LVSI/LVI) positivity after exclusion of studies with available-case denominators or potential cohort overlap, shown as a random-effects forest plot. LVSI, lymphovascular space invasion; LVI, lymphovascular invasion; OR, odds ratio; CI, confidence interval.
