# Supplementary figures and images for: Clinicopathological features and survival outcomes of HPV-independent versus HPV-associated cervical adenocarcinoma: a Systematic Review and meta-analysis
Source: Front Oncol. 2026 May 8;16:1837655. doi: 10.3389/fonc.2026.1837655 (PMC13194532; doi:10.3389/fonc.2026.1837655)

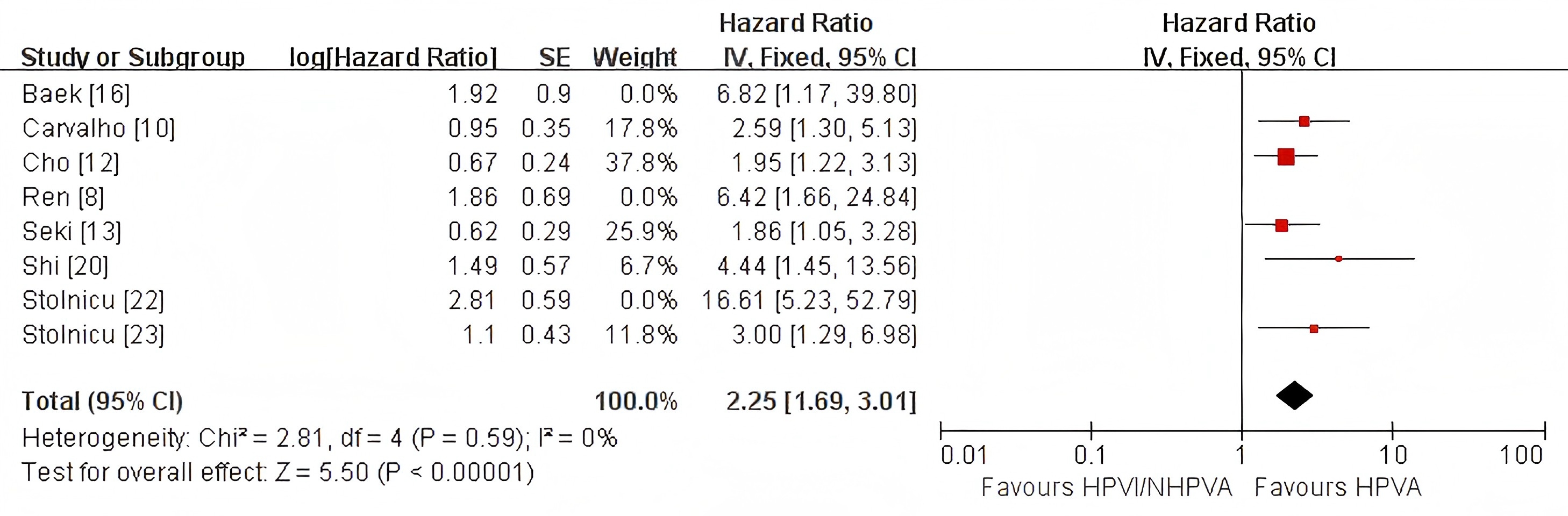

Supplement: Supplementary Figure 1 — Sensitivity analysis of overall survival (OS) after exclusion of studies requiring additional assumptions for effect extraction or harmonization, shown as a fixed-effect forest plot. OS, overall survival; HR, hazard ratio; CI, confidence interval. [file Image1.jpeg]

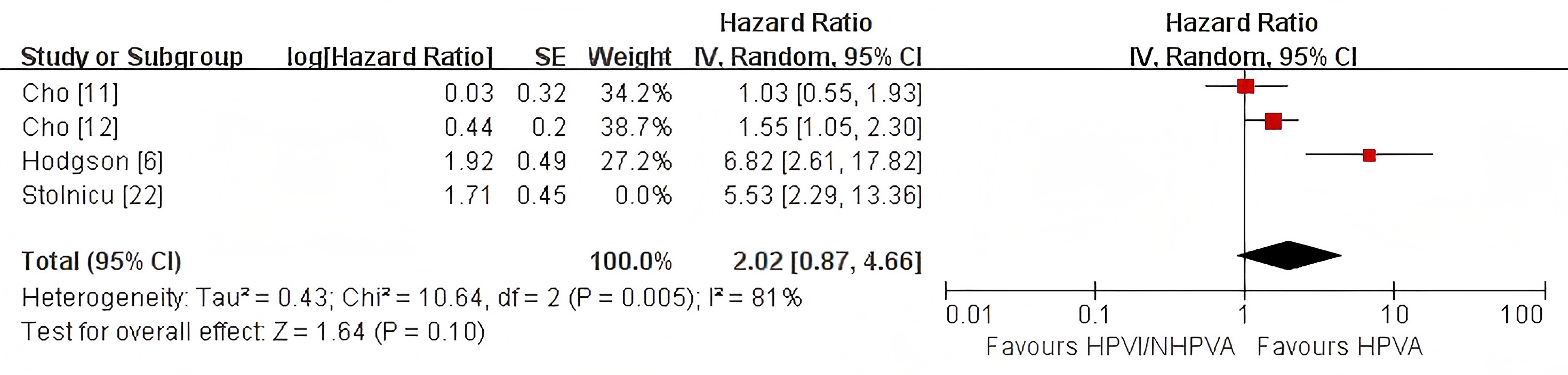

Supplement: Supplementary Figure 2 — Sensitivity analysis of disease-free survival (DFS) after exclusion of the study requiring reciprocal transformation to align the reported comparison with the prespecified analytic direction, shown as a random-effects forest plot. DFS, disease-free survival; HR, hazard ratio; CI, confidence interval. [file Image2.jpeg]

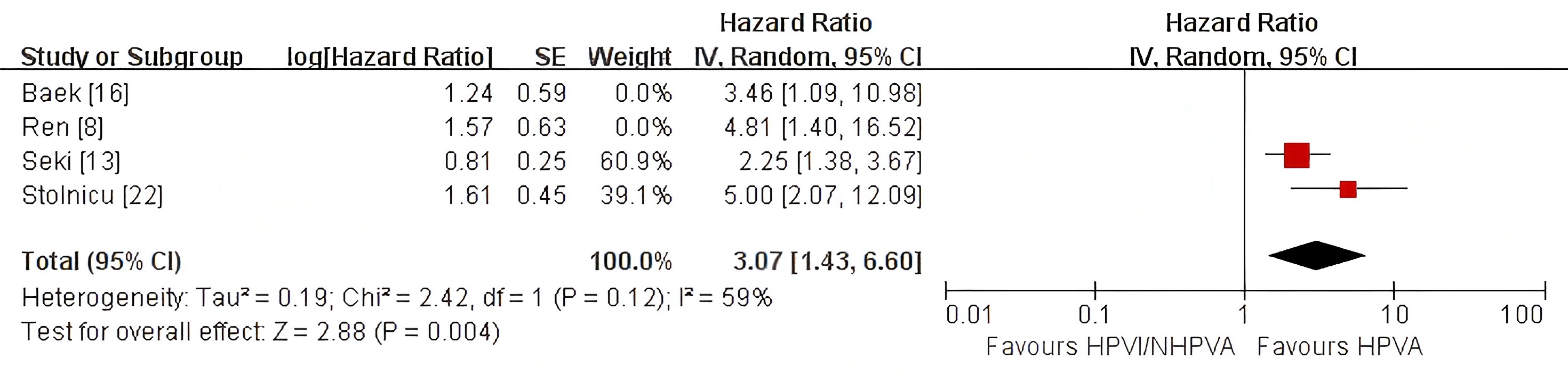

Supplement: Supplementary Figure 3 — Sensitivity analysis of progression-free survival (PFS) after exclusion of studies with concerns regarding reporting direction or figure-derived extraction, shown as a random-effects forest plot. PFS, progression-free survival; HR, hazard ratio; CI, confidence interval. [file Image3.jpeg]

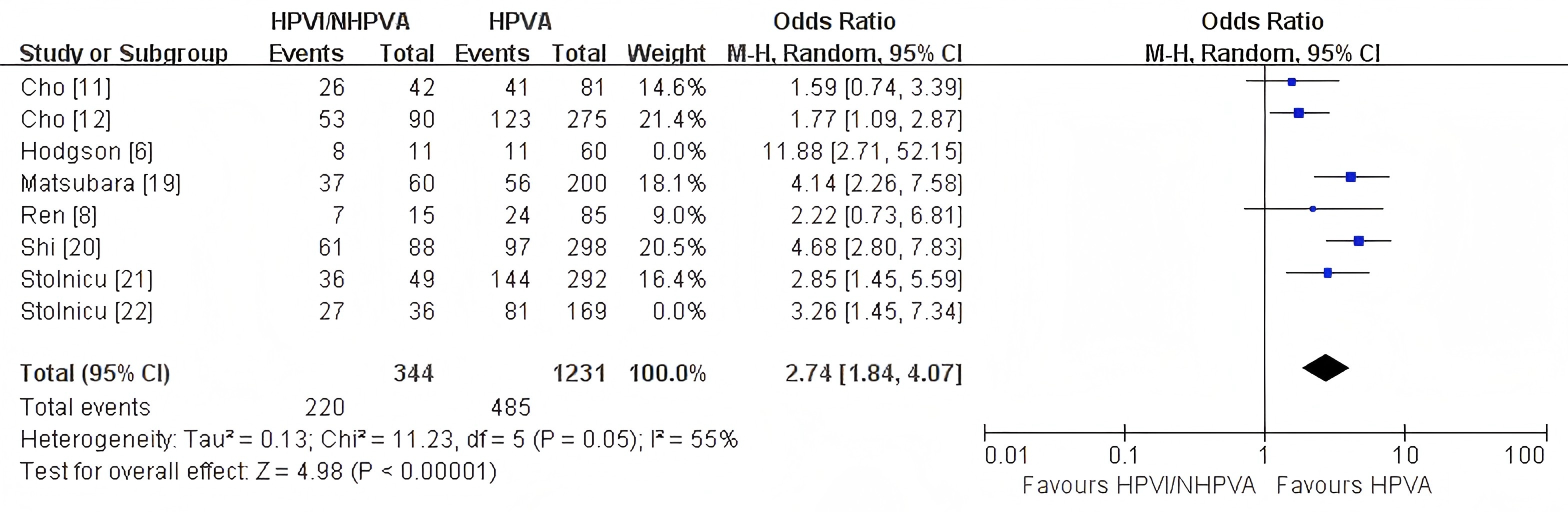

Supplement: Supplementary Figure 4 — Sensitivity analysis of lymphovascular space invasion/lymphovascular invasion (LVSI/LVI) positivity after exclusion of studies with available-case denominators or potential cohort overlap, shown as a random-effects forest plot. LVSI, lymphovascular space invasion; LVI, lymphovascular invasion; OR, odds ratio; CI, confidence interval. [file Image4.jpeg]
