# Supplementary material for: Clinicopathological features and survival outcomes of HPV-independent versus HPV-associated cervical adenocarcinoma: a Systematic Review and meta-analysis
Source: Front Oncol. 2026 May 8;16:1837655. doi: 10.3389/fonc.2026.1837655 (PMC13194532; doi:10.3389/fonc.2026.1837655)
Supplement: Supplementary file 5 [file Table1.docx]

### Supplementary Methods: Literature search strategy

We systematically searched PubMed, Embase, Scopus, Web of Science Core Collection, and the Cochrane Library to identify studies evaluating clinicopathological features and survival outcomes of HPV-independent versus HPV-associated cervical adenocarcinoma. The searches were performed from database inception to 01 March 2026. In addition, the reference lists of all included studies and relevant reviews were manually screened to identify further eligible studies. The detailed search strategies for each database are provided below.

#### 1. PubMed

**Database:** PubMed (MEDLINE)
**Date range:** from database inception to 01 March 2026
**Search fields:** Title/Abstract and related indexing terms

**Search strategy:**

(("endocervical adenocarcinoma" OR "cervical adenocarcinoma" OR "adenocarcinoma of the cervix" OR "adenocarcinoma of uterine cervix")
AND
("HPV-independent" OR "HPV independent" OR "non-HPV-associated" OR "non HPV-associated" OR "non-HPVA" OR NHPVA OR HPVI OR "HPV-associated" OR "HPV associated" OR HPVA OR "HPV-related" OR "HPV related" OR "HPV-negative" OR "HPV positive" OR IECC OR "International Endocervical Adenocarcinoma Criteria and Classification" OR "WHO 2020" OR "WHO classification")
AND
(clinicopatholog* OR prognos* OR survival OR outcome* OR recurren* OR metasta* OR response))

#### 2. Embase

**Database:** Embase
**Date range:** from database inception to 01 March 2026

**Search strategy:**

('endocervical adenocarcinoma' OR 'cervical adenocarcinoma' OR 'adenocarcinoma of the cervix' OR 'adenocarcinoma of uterine cervix')
AND
('HPV-independent' OR 'HPV independent' OR 'non-HPV-associated' OR 'non HPV-associated' OR 'non-HPVA' OR NHPVA OR HPVI OR 'HPV-associated' OR 'HPV associated' OR HPVA OR 'HPV-related' OR 'HPV related' OR 'HPV-negative' OR 'HPV positive' OR IECC OR 'International Endocervical Adenocarcinoma Criteria and Classification' OR 'WHO 2020' OR 'WHO classification')
AND
(clinicopatholog* OR prognos* OR survival OR outcome* OR recurren* OR metasta* OR response)

#### 3. Web of Science Core Collection

**Database:** Web of Science Core Collection
**Search field:** Topic (TS)
**Date range:** from database inception to 01 March 2026

**Search strategy:**

TS=(("endocervical adenocarcinoma" OR "cervical adenocarcinoma" OR "adenocarcinoma of the cervix" OR "adenocarcinoma of uterine cervix")
AND
("HPV-independent" OR "HPV independent" OR "non-HPV-associated" OR "non HPV-associated" OR "non-HPVA" OR NHPVA OR HPVI OR "HPV-associated" OR "HPV associated" OR HPVA OR "HPV-related" OR "HPV related" OR "HPV-negative" OR "HPV positive" OR IECC OR "International Endocervical Adenocarcinoma Criteria and Classification" OR "WHO 2020" OR "WHO classification")
AND
(clinicopatholog* OR prognos* OR survival OR outcome* OR recurren* OR metasta* OR response))

#### 4. Scopus

**Database:** Scopus
**Search field:** TITLE-ABS-KEY
**Date range:** from database inception to 01 March 2026

**Search strategy:**

TITLE-ABS-KEY(("endocervical adenocarcinoma" OR "cervical adenocarcinoma" OR "adenocarcinoma of the cervix" OR "adenocarcinoma of uterine cervix")
AND
("HPV-independent" OR "HPV independent" OR "non-HPV-associated" OR "non HPV-associated" OR "non-HPVA" OR NHPVA OR HPVI OR "HPV-associated" OR "HPV associated" OR HPVA OR "HPV-related" OR "HPV related" OR "HPV-negative" OR "HPV positive" OR IECC OR "International Endocervical Adenocarcinoma Criteria and Classification" OR "WHO 2020" OR "WHO classification")
AND
(clinicopatholog* OR prognos* OR survival OR outcome* OR recurren* OR metasta* OR response))

#### 5. Cochrane Library

**Database:** Cochrane Library
**Date range:** from database inception to 01 March 2026

**Search strategy:**

(("endocervical adenocarcinoma" OR "cervical adenocarcinoma" OR "adenocarcinoma of the cervix" OR "adenocarcinoma of uterine cervix")
AND
("HPV-independent" OR "HPV independent" OR "non-HPV-associated" OR "non HPV-associated" OR "non-HPVA" OR NHPVA OR HPVI OR "HPV-associated" OR "HPV associated" OR HPVA OR "HPV-related" OR "HPV related" OR "HPV-negative" OR "HPV positive" OR IECC OR "International Endocervical Adenocarcinoma Criteria and Classification" OR "WHO 2020" OR "WHO classification")
AND
(clinicopatholog* OR prognos* OR survival OR outcome* OR recurren* OR metasta* OR response))
