# Supplementary material for: Clinicopathological features and survival outcomes of HPV-independent versus HPV-associated cervical adenocarcinoma: a Systematic Review and meta-analysis
Source: Front Oncol. 2026 May 8;16:1837655. doi: 10.3389/fonc.2026.1837655 (PMC13194532; doi:10.3389/fonc.2026.1837655)
Supplement: Supplementary file 6 [file Table2.docx]

| Supplementary Table 1. Detailed data extraction of studies comparing HPV-independent and HPV-associated cervical/endocervical adenocarcinoma | | | | | | | | | | | | | | | | | | | | | | | | | | | | | | | | | | | | | | | | | | | |
| --- | --- | --- | --- | --- | --- | --- | --- | --- | --- | --- | --- | --- | --- | --- | --- | --- | --- | --- | --- | --- | --- | --- | --- | --- | --- | --- | --- | --- | --- | --- | --- | --- | --- | --- | --- | --- | --- | --- | --- | --- | --- | --- | --- |
| Authors | Year | Country | Study design | Population / disease entity | Total (n) | HPVI (n) | HPVA (n) | Other (n) | Age overall | Age HPVI | Age HPVA | Stage range | Classification framework | p16 used | HPV DNA used | HPV RNA ISH used | Definition of HPVI | Definition of HPVA | Main HPVI subtype(s) | Main HPVA subtype(s) | Treatment modality | Advanced stage | Tumor size | Grade / differentiation | LVSI | Deep stromal invasion | Lymph node metastasis | Parametrial invasion | Margin status | Local recurrence | Distant metastasis | OS HR | OS 95% CI | DFS HR | DFS 95% CI | PFS HR | PFS 95% CI | RFS HR | RFS 95% CI | Response data | Multivariable analysis | Adjusted variables | Study period |
| Baek et al. [16] | 2025 | Korea | Retrospective cohort | Endocervical adenocarcinoma | 40 | 18 | 22 | 0 | NR | 52 (40–78) | 49 (30–71) | HPVA：IB 7，IIB–IIIB 3，IIIC1 7，IIIC2 5，IVA 0 HPVI：IB 0，IIB–IIIB 6，IIIC1 9，IIIC2 2，IVA 1 | IECC and 2020 WHO morphology-based classification | NR | NR | NR | tumors without easily identifiable apical mitotic activity/apoptotic bodies, or only focal/equivocal HPVA features at high-power magnification | tumors with clearly identifiable apical mitotic figures and apoptotic bodies | gastric 14/18 (77.8%), NOS 3/18 (16.7%), clear cell 1/18 (5.6%) | usual 18/22 (81.8%), mucinous ISM 3/22 (13.6%), mucinous NOS 1/22 (4.5%) | Definitive CCRT | Reported | HPVA: 5.2 (2.7–7.3) cm HPVI: 5.5 (3.9–8.5) cm | NR | NR | NR | HPVA: 12/22 (54.5%) HPVI: 13/18 (72.2%) | HPVA: 10/22 (45.5%) HPVI: 17/18 (94.4%) | NR | HPVA：LRR 8/22，DM 2/22，LRR+DM 1/22 HPVI：LRR 5/18，DM 6/18，LRR+DM 5/18*** | Reported*** | 6.83 | 1.17–39.80 | NR | NR | 3.44 | 1.09–10.81 | NR | NR | HPVA 18/22 (81.8%) vs HPVI 5/18 (27.8%) | Yes | CR model: HPV status, parametrial invasion, regressed TV during RT Survival models: HPV status, tumor volume, parametrial invasion, regressed TV during RT | NR |
| Ben-Mussa et al. [17] | 2025 | Northern Ireland, United Kingdom | Population-based retrospective cohort | Primary cervical adenocarcinoma | 146 | 16 | 130 | 0 | 44 (24–84) | 62.5 (31–84) | 43 (24–82) | FIGO 2018 IA1–IVB | Current WHO HPV-associated vs HPV-independent framework with specialist pathology review | Yes | No | No | WHO-defined HPV-independent morphology on specialist pathology review | WHO-defined HPV-associated morphology on specialist pathology review, supported by p16 | Gastric type 9/16 (56.3%); clear cell 5/16 (31.3%); mesonephric 1/16 (6.3%); serous 1/16 (6.3%) | Usual type 120/130 (92.3%); ISMC 6/130 (4.6%); signet ring 3/130 (2.3%); clear cell 1/130 (0.8%) | NR | Reported (HPVI more advanced; article has internal Stage I discrepancy, abstract/text 97/130 vs table-based 96/130 for HPVA) | NR | NR | NR | NR | NR | NR | NR | NR | NR | NR | NR | NR | NR | NR | NR | NR | NR | No | No | NR | 2015–2023 |
| Carvalho et al. [10] | 2022 | Brazil | Retrospective longitudinal study | Endocervical adenocarcinoma | 140* | 17 | 100 | 23 | NR | 57.3 ± 17.2 | 47.8 ± 12.7 | FIGO 2011, analyzed as Stage I vs II+ | 2020 WHO / IECC morphology-based reclassification | Yes (equivocal cases only) | No | No | Mitoses and apoptosis not abundant or not easily identified | Presence of apical mitotic figures and apoptotic bodies | HPVI NOS 9/17 (52.9%); clear cell 5/17 (29.4%); mesonephric 2/17 (11.7%); gastric 1/17 (5.8%) | Usual 69/100 (69.0%); mucinous 16/100 (16.0%); iSMILE 10/100 (10.0%); HPVA NOS 5/100 (5.0%) | Mixed | Stage II+: HPVI 14/17 (82.3%) vs HPVA 48/100 (48.0%) | NR | NR | NR | NR | NR | NR | NR | NR | NR | 2.59 | 1.30–5.18 | NR | NR | NR | NR | NR | NR | NR | Yes | FIGO stage; age | 2013–2020 |
| Cho et al. [11] | 2022 | Korea | Retrospective cohort | Endocervical adenocarcinoma | 123 | 42 | 81 | 0 | 48 (16–67) | 48 (16–67) | 48 (28–76) | Clinical FIGO 2018 I–III; pathological FIGO 2018 IB–IIIC2 | IECC criteria and updated WHO classification | NR | NR | NR | Endocervical ADC with no readily identifiable apical mitotic activity/apoptotic bodies, or only focal/equivocal HPVA features at high-power magnification | Endocervical ADC with easily identified apical mitotic figures and apoptotic bodies | Gastric 30/42 (71.4%); clear cell 4/42 (9.5%); mesonephric 3/42 (7.1%); serous 3/42 (7.1%); NOS 2/42 (4.8%) | Usual 60/81 (74.0%); mucinous ISM 10/81 (12.3%); mucinous intestinal 8/81 (9.9%); mucinous NOS 3/81 (3.7%) | Radical hysterectomy + adjuvant RT ± concurrent chemotherapy | Reported | HPVI: 3.85 (0.7–10.0) cm; HPVA: 4.1 (0.8–11.0) cm | NR | HPVI: 26/42 (61.9%); HPVA: 41/81 (50.6%) | HPVI >50%: 31/42 (73.8%); HPVA >50%: 60/81 (74.1%) | Reported; subgroup counts internally inconsistent in source | HPVI: 18/42 (42.9%); HPVA: 14/81 (17.3%) | HPVI positive VRM: 8/42 (19.0%); HPVA: 3/81 (3.7%) | HPVI: 8/42 (19.0%); HPVA: 7/81 (8.6%) | HPVI: 20/42 (47.6%); HPVA: 22/81 (27.2%) | NR | NR | 1.03 | 0.550–1.927 | NR | NR | NR | NR | NR | Yes | FIGO stage; vaginal resection margin; adjuvant treatment | 2001–2018 |
| Cho et al. [12] | 2023 | Korea | Retrospective multi-institutional cohort | Endocervical adenocarcinoma | 365 | 90 | 275 | 0 | 47 (16–76) | 48 (16–75) | 47 (27–76) | FIGO 2018 IB–IIIC2 | 2020 WHO / IECC morphology-based classification | NR | NR | NR | Tumors with no readily identifiable apical mitotic activity/apoptotic bodies, or only focal/equivocal HPV-related features appreciable at high-power magnification | Tumors with apical mitotic figures and apoptotic bodies readily identifiable at scanning magnification | Gastric 57/90 (63.3%); clear cell 9/90 (10.0%); serous 6/90 (6.7%); mesonephric 4/90 (4.4%); NOS 14/90 (15.6%) | Usual 217/275 (78.9%); mucinous-intestinal 19/275 (6.9%); mucinous-ISM 13/275 (4.7%); mucinous-SRC 3/275 (1.1%); mucinous-NOS 23/275 (8.4%) | Radical hysterectomy + pelvic LN dissection; adjuvant treatment mixed (none / RT alone / CCRT / sequential CTx-RT / CTx alone) | Reported (if defined as IIB–IIIC2: HPVI 52/90 [57.8%] vs HPVA 107/275 [38.9%]) | HPVI: 3.7 (0.7–10.0) cm; HPVA: 3.0 (0.3–11.0) cm | NR | HPVI: 53/90 (58.9%); HPVA: 123/275 (44.7%) | HPVI >50%: 68/90 (75.6%); HPVA >50%: 178/275 (64.7%) | Reported as para-aortic LN metastasis: HPVI 32/90 (35.6%); HPVA 98/275 (35.6%) | HPVI: 34/90 (37.8%); HPVA: 52/275 (18.9%) | HPVI positive margin 15/90 (16.7%); HPVA 12/275 (4.4%) | HPVI 23/90 (25.6%); HPVA 30/275 (10.9%) | HPVI 30/90 (33.3%); HPVA 48/275 (17.5%) | 1.959 | 1.230–3.121 | 1.559 | 1.062–2.289 | NR | NR | NR | NR | NR | Yes | Histology; FIGO stage; invasion depth; LVSI; resection margin involvement; adjuvant RT; adjuvant CCRT | 2001–2018 |
| He et al. [18] | 2025 | China | Retrospective single-center cohort | Primary cervical adenocarcinoma | 332 | 47 | 285 | 0 | 52 (mean; range 27–82) | 56 (median) | 51 (median) | FIGO stage I–IV (table counts internally incomplete) | 2020 WHO classification with IECC-based grouping into HPV-Ind-CA vs HPV-CA | NR | NR | NR | WHO/IECC-defined HPV-independent cervical adenocarcinoma, including gastric-type, clear cell, endometrioid, and invasive adenocarcinoma NOS | WHO/IECC-defined HPV-related cervical adenocarcinoma, including usual type, mucinous intestinal type, ISMC, and mucinous adenocarcinoma NOS | Gastric-type 30; invasive adenocarcinoma NOS 7; endometrioid 6; clear cell 4 | Usual type 255; mucinous adenocarcinoma NOS 20; intestinal-type 9; ISMC 1 | Mixed (surgery / adjuvant radiotherapy / adjuvant chemotherapy) | Reported; HPV-Ind-CA presented at more advanced stage, but stage table counts are internally incomplete | Reported (MD categories: <2, ≥2–<4, ≥4 cm; counts internally incomplete) | High/Moderate/Low reported: HPV-Ind-CA 12/18/17; HPV-CA 26/196/63 | NR | NR | NR | NR | NR | NR | NR | NR | NR | NR | NR | NR | NR | NR | NR | Yes (CR/SD/PD reported) | Yes | Overall model: age, FIGO stage, HPVI status, CA125, CA199; HPV-Ind-CA subgroup: differentiation, CA199; HPV-CA subgroup: age, FIGO stage, HPVI status, CA125, CA199; no direct adjusted HR for HPV-Ind-CA vs HPV-CA classification | 2012.10–2023.07 |
| Hodgson et al. [6] | 2019 | Canada | Retrospective single-center cohort | Endocervical adenocarcinoma | 87 | 16 | 71 | 0 | NR | 58 (17–69) | 40 (22–80) | FIGO stage I–IV (stage data available for n=83) | IECC morphology-based classification | Yes (ancillary correlation only) | Yes (HPV ISH/PCR in subset; ancillary only) | NR | Tumors with mitoses and apoptosis not abundant or not easily identified | Tumors with easily identifiable apical mitotic figures and apoptotic bodies | Gastric 12/16 (75%); mesonephric 2/16 (13%); clear cell 2/16 (12%) | Usual 51/71 (72%); mucinous NOS 10/71 (14%); ISMC 8/71 (11%); mucinous intestinal 2/71 (3%) | Mixed (surgical resection / chemoradiation) | FIGO stage II–IV: HPVI 12/16 vs HPVA 7/67 (available cases only) | Horizontal extent: HPVI 21.0 mm (2.0–45.0) vs HPVA 9.0 mm (2.0–50.0) | NR | LVSI present: HPVI 8/11 vs HPVA 11/60 (available cases only) | Depth of invasion: HPVI 11.0 mm (4.0–30.0) vs HPVA 4.5 mm (1.0–25.0) | Lymph node involvement present: HPVI 2/8 vs HPVA 2/34 (available cases only) | NR | NR | NR | NR | NR | NR | 6.8 | 2.6–17.8 | NR | NR | NR | NR | No | Yes | IECC group; horizontal extent; depth of invasion; LVSI; stage (multivariable DFS model reported, but no detailed table) | 2002.01–2017.12 |
| Matsubara et al. [19] | 2025 | Japan | Multicenter retrospective cohort | Cervical adenocarcinoma | 264* | 60 | 201 | 3 | 51 ± 14 | 60 ± 13 | NR (HPVA overall not separately tabulated; subgroup means around 47–48 years) | Preoperative FIGO 2008 stage I–II | Morphology-based HPVA vs HPVI classification using the international group criteria and WHO 5th edition; HPVA further stratified by Silva / binary Silva | No | No | No | Morphology-based HPV-independent adenocarcinoma according to the international group criteria / WHO 5th edition | Morphology-based HPV-associated adenocarcinoma according to the international group criteria / WHO 5th edition | NR | NR | Surgery as initial treatment; adjuvant therapy = none / adjuvant chemotherapy / adjuvant RT / adjuvant CCRT | Preoperative stage II: HPVI 20/60 (33.3%) vs HPVA 23/201 (11.4%) | MRI tumor size categories reported (≤2 cm / 2–4 cm / >4 cm); HPVI enriched in larger tumors | NR | LVSI present: HPVI 37/60 (61.7%) vs HPVA 56/200 (28.0%) | MRI stromal invasion ≥50%: HPVI 42/54 (77.8%) vs HPVA 54/176 (30.7%) | Lymph node metastasis: HPVI 21/55 (38.2%) vs HPVA 14/177 (7.9%) | NR | NR | Local recurrence: HPVI 8/59 (13.6%) vs HPVA 11/201 (5.5%) | Distant metastasis: HPVI 9/59 (15.3%) vs HPVA 4/201 (2.0%) | NR | NR | NR (no direct overall HPVI vs pooled HPVA DFS HR; three-tier model reported instead) | NR | NR | NR | NR | NR | No | Yes | Three-tier system (binary Silva-classified HPVA/HPVI); MRI tumor size; age; sensitivity model additionally adjusted for surgical procedure and adjuvant therapy | 2011.01–2020.12 |
| Ren et al. [8] | 2021 | Canada | Retrospective single-center resection cohort | Endocervical adenocarcinoma | 100 | 15 | 85 | 0 | 47 (11), mean ± SD; median 45 (39–53) | 57 (14), mean ± SD; median 52 (49–65) | 45 (10), mean ± SD; median 44 (38–51) | FIGO 2019 stage I–III | IECC using histology + p16 IHC + HPV RNA ISH | Yes | No | Yes | Presence of classic HPVA histomorphology with apical mitotic figures and apoptotic bodies visible at scanning magnification | Tumors lacking classic HPVA histomorphology, substratified as gastric/clear cell/endometrioid/mesonephric etc. | Gastric 9/15 (60%); mesonephric 4/15 (27%); clear cell 1/15 (7%); NOS 1/15 (7%) | Usual 63/85 (74%); villoglandular 4/85 (5%); mucinous intestinal 3/85 (4%); mucinous NOS 15/85 (18%) | Resected cohort with/without postsurgical adjuvant treatment | Stage II/III: HPVI 5/15 (33.3%) vs HPVA 7/85 (8.2%) | HPVI 2.4 ± 1.1 cm; HPVA 1.6 ± 1.2 cm | Grade 1/2/3: HPVI 6/6/1; HPVA 43/37/5 | LVSI positive: HPVI 7/15 (46.7%) vs HPVA 24/85 (28.2%) | NR | Overall 4 cases; by HPV group not clearly tabulated | NR | Positive margins: HPVI 3/15 (20.0%) vs HPVA 2/83 (2.4%) | NR | NR | Reported in figure only; likely HPVA vs HPVI = 0.156 | 0.040–0.606 | NR | NR | Reported in figure only; likely HPVA vs HPVI = 0.208 | 0.063–0.753 | NR | NR | No | Yes | Model 1: HPV, age, margin, tumor size, Silva pattern, grade, stage, LVI, adjuvant treatment; Model 2: HPV, age, stage, treatment | NR |
| Seki et al. [13] | 2023 | Japan | Multicenter retrospective cohort | Locally advanced cervical adenocarcinoma | 151 | 48 | 103 | 0 | NR (continuous age not separately reported) | NR (>50 years: 32/48, 66.7%) | NR (>50 years: 41/103, 39.8%) | FIGO 2018 IB3–IIIC1 | 2020 WHO morphology-based HPVi vs HPVa classification with central pathology review; p16/p53 used as support; no molecular HPV testing | Yes | No | No | Morphology-based HPV-independent adenocarcinoma per 2020 WHO/IECC, supported by p16/p53 | Morphology-based HPV-associated adenocarcinoma per 2020 WHO/IECC, supported by p16/p53 | Gastric type dominant (~90%); clear cell carcinoma 4 cases | Usual type 84%; mucinous carcinoma NOS 15% | Locally advanced cohort treated regardless of primary modality; surgery 43/48 vs 89/103, RT/CCRT 5/48 vs 14/103; NAC and adjuvant therapy reported | Advanced stage within cohort (IIB–IIIC1): HPVI 35/48 (72.9%) vs HPVA 50/103 (48.5%) | Tumor diameter >40 mm: HPVI 16/48 (33.3%) vs HPVA 18/103 (17.5%); unknown 2 vs 4 | NR | NR | NR | Radiologic lymphadenopathy: HPVI 13/48 (27.1%) vs HPVA 18/103 (17.5%)**** | Parametrial invasion: HPVI 26/48 (54.2%) vs HPVA 28/101 (27.7%), with 2 HPVA missing | NR | NR | NR | 1.85 | 1.05–3.25 | NR | NR | 2.24 | 1.38–3.65 | NR | NR | Subgroup only: 5 HPVi definitive RT/CCRT cases all responded, 3 complete responses | Yes | PFS model: age, lymphadenopathy, parametrial invasion, treatment method, histological type; OS model: lymphadenopathy, parametrial invasion, treatment method, histological type; FIGO stage excluded due to collinearity | 2004–2009 |
| Shi et al. [20] | 2022 | China | Retrospective single-center resection cohort | Cervical adenocarcinoma | 402* | 88 | 298 | 16 | NR | 50.1 ± 11.4 | 45.5 ± 9.2 | FIGO I–IV | WHO 2020 / IECC morphology-based classification | Yes | No | Yes | Morphology-based HPV-independent adenocarcinoma per WHO 2020/IECC, lacking classic HPV-related apical mitoses/apoptotic bodies | Morphology-based HPV-related adenocarcinoma per WHO 2020/IECC, with readily identifiable apical mitotic figures and apoptotic bodies | Gastric type 73/88 (83.0%); clear cell 10/88 (11.4%); endometrioid 4/88 (4.5%); mesonephric 1/88 (1.1%) | Usual type 224/298 (75.2%); mucinous NOS 27/298 (9.1%); ISMC 26/298 (8.7%); villoglandular 14/298 (4.7%); mucinous intestinal 6/298 (2.0%); signet-ring cell 1/298 (0.3%) | Resected specimens (conisation / trachelectomy / hysterectomy), with variable postoperative management | FIGO II–IV: HPVI 45/88 vs HPVA 36/296 (HPVA stage data slightly incomplete) | HPVI 3.0 ± 0.8 cm; HPVA 2.2 ± 0.7 cm | NR | LVSI positive: HPVI 61/88 (69.3%) vs HPVA 97/298 (32.6%) | Depth of invasion >2/3 cervical wall: HPVI 64/88 vs HPVA 97/296 (available cases only) | NR | NR | NR | NR | NR | 4.422 | 1.437–13.613 | NR | NR | NR | NR | 4.371 | 1.602–11.925 | No | Yes | OS model: age (>46), WHO classification, FIGO stage, LVSI; tumour recurrence model: WHO classification, FIGO stage, mutant-type p53 expression, LVSI | 2004.01–2019.12 |
| Stolnicu et al. [21] | 2018 | International | Multicenter retrospective cohort | Invasive adenocarcinomas of the endocervix | 370* | 55 | 306 | 9 | NR | 55 (43–67) | 42 (36–52) | FIGO I–IV | IECC morphology-based classification | Yes | No | Yes | No easily identifiable apical mitotic activity/apoptotic bodies at scanning magnification; focal/equivocal limited HPVA features at high power classified as NHPVA | Apical mitotic figures and apoptotic bodies appreciable at scanning magnification; if not obvious, brief high-power review used | Gastric 37; clear cell 11; endometrioid 4; serous 2; mesonephric 1 | Usual 271; mucinous NOS 11; mucinous intestinal 11; signet ring 1; iSMILE 9; villoglandular 3 | Surgical/excision cohort only; no neoadjuvant therapy | Stage II–IV: HPVI 24/50 vs HPVA 26/287 | HPVI 38 mm (21–45) vs HPVA 21 mm (10–35) | NR | NR | NR | NR | NR | NR | NR | NR | NR | NR | NR | NR | NR | NR | NR | NR | No | No | NR | NR |
| Stolnicu et al. [22] | 2019 | International | Multicenter retrospective cohort | Endocervical adenocarcinoma | 341 | 49 | 292 | 0 | NR | NR | NR | FIGO I–IV | IECC morphology-based classification | No | No | Yes | No easily identifiable apical mitotic activity/apoptotic bodies at scanning magnification; focal/equivocal limited HPVA features at high power classified as NHPVA | Apical mitotic figures and apoptotic bodies appreciable at scanning magnification | Gastric 33/49; clear cell 6/49; adenocarcinoma NOS 4/49; endometrioid 3/49; serous 2/49; mesonephric 1/49 | Usual 259/292; mucinous NOS 10/292; mucinous intestinal 9/292; iSMILE 9/292; adenocarcinoma NOS 5/292 | Surgical/excision cohort only; no neoadjuvant therapy | FIGO stage II–IV: HPVI 23/44 vs HPVA 28/274 (available cases only) | HPVI 40 mm vs HPVA 21 mm (median) | NR | LVI/LVSI positive: HPVI 36/49 (73.5%) vs HPVA 144/292 (49.3%) | NR | LNM positive: HPVI 15/49 (30.6%) vs HPVA 44/292 (15.1%) | NR | NR | NR | NR | 1.1 | 0.6–2.0 | NR | NR | NR | NR | 1.2 | 0.7–1.9 | No | Yes | Stage | NR |
| Stolnicu et al. [23] | 2021 | International | Multicenter retrospective cohort | Endocervical adenocarcinoma | 205 | 36 | 169 | 0 | NR | NR | NR | FIGO I–IV | IECC morphology-based classification with high-risk HPV RNA ISH confirmation | NR | No | Yes | No easily identifiable apical mitotic activity/apoptotic bodies on scanning magnification; focal/equivocal limited HPV-associated features at high power still classified as NHPVA | Apical mitotic figures and apoptotic bodies appreciable at scanning magnification | Gastric 24; clear cell 7; endometrioid 2; serous 1; mesonephric 1; adenocarcinoma NOS 1 | Usual 147; mucinous NOS 3; mucinous intestinal 4; iSMILE 9; villoglandular 2; adenocarcinoma NOS 4 | Surgical/excision cohort; surgery alone vs surgery + adjuvant treatment; neoadjuvant therapy excluded | FIGO stage II–IV: HPVI 18/36 vs HPVA 17/169 | NR (reported by subtype only, not pooled HPVA/NHPVA aggregate) | NR | LVI/LVSI positive: HPVI 27/36 (75.0%) vs HPVA 81/169 (47.9%) | NR | LNM positive: HPVI 7/36 (19.4%) vs HPVA 24/169 (14.2%) | NR | NR | Local recurrence: HPVI 8/36 (22.2%) vs HPVA 15/169 (8.9%) | Distant metastasis: HPVI 6/36 (16.7%) vs HPVA 12/169 (7.1%) | 16.67** | 5.88–58.82 | 5.56** | 2.44–14.29 | 5** | 2.13–12.50 | NR | NR | No | Yes | Covariates included: age, FIGO stage, grade, tumor size, Silva pattern; Silva pattern excluded in NHPVA-only model | NR |
| Stolnicu et al. [3] | 2018 | International | International multicenter retrospective cohort | Endocervical adenocarcinoma | 464* | 52 | 329 | 83 | NR | NR | NR | FIGO 2018 stage IB1–IB3 | IECC morphology-based HPV-associated vs HPV-independent classification | NR | No | NR | HPV-independent endocervical adenocarcinoma lacking classic HPV-associated morphology | HPV-associated endocervical adenocarcinoma with apical mitotic figures and apoptotic bodies appreciable at scanning magnification | NR | NR | Surgical cohort only (conization / trachelectomy / hysterectomy), no neoadjuvant therapy | No (stage IB cohort only) | Reported, primarily stage-stratified rather than directly tabulated by HPV group | Reported, primarily stage-stratified rather than directly tabulated by HPV group | Reported, primarily stage-stratified rather than directly tabulated by HPV group | NR | Reported, primarily stage-stratified rather than directly tabulated by HPV group | NR | NR | Reported, primarily stage-stratified rather than directly tabulated by HPV group | Reported, primarily stage-stratified rather than directly tabulated by HPV group | 3 | 1.29–6.99 | NR | NR | NR | NR | 2.31 | 1.02–5.46 | No | Yes | FIGO stage; precursor lesions; HPV status; Silva pattern; tumor grade; LVSI; lymph node metastases | 1985–2019 |
| Williams et al. [24] | 2026 | USA | Retrospective cohort | Invasive endocervical adenocarcinoma | 144 | 34 | 110 | 0 | 46.8 ± 12.5; median 44.5 (38.0–54.0) | 54.6 ± 14.2; median 53.5 (46.0–61.0) | 44.4 ± 10.9; median 42.0 (37.0–50.0) | 2009 FIGO stage IA–IV | Integrated IECC-based classification using morphology with available digene HPV results and ancillary immunohistochemistry | Yes (when available) | Yes (clinical digene HPV testing prior to diagnosis, not uniform) | No | Non-HPV-associated invasive endocervical adenocarcinoma by integrated IECC morphology + available clinical/ancillary support | HPV-associated invasive endocervical adenocarcinoma by integrated IECC morphology + available clinical/ancillary support | Clear cell 4 (11.8% of NHPVA); endometrioid 8 (23.5%); mucinous 19 (55.9%); serous 3 (8.8%) | Usual-type 110 (100%) | Mixed primary treatment modality: surgery alone 21/34 vs 79/110; chemoradiation 2/34 vs 15/110; multimodal 11/34 vs 16/110 | Stage II–IV: HPVI/NHPVA 9/34 (26.5%) vs HPVA 15/110 (13.6%) | NR | NR | NR | NR | NR | NR | NR | NR | NR | NR | NR | NR | NR | NR | NR | NR | NR | No | No | NR | 2011–2017 |
| Yasutake et al. [25] | 2024 | Japan | Retrospective cohort | Invasive endocervical adenocarcinoma | 103* | 25 | 78 | 0 | 45 (28–83), median for overall ECA cohort | NR | NR | FIGO 2018 stage I–IV | WHO 5th morphology-based classification with p16 IHC, Rb IHC, and HR-HPV mRNA ISH | Yes | No | Yes | HPV-independent adenocarcinoma defined as invasive cancer according to the fifth WHO classification, lacking HR-HPV by mRNA ISH | HPV-associated adenocarcinoma defined as invasive cancer according to the fifth WHO classification, positive for HR-HPV by mRNA ISH | Gastric type 14; clear cell type 10; NOS 1 | Usual type 67; mucinous type 11 | Mixed biopsy/resection cohort; patients subsequently treated with surgery or radiation | Reported (HPV-positive associated with lower stage) | NR | NR | NR | NR | Reported (HPV-positive associated with lower lymph node metastasis rate) | NR | NR | Reported (overall recurrence rate lower in HPV-positive) | NR | NR | NR | NR | NR | NR | NR | NR | NR | No | Yes | Reported in Supporting Table S4; Cox model included age, stage, lymph node metastasis, HR-HPV status, and Rb pattern at least in univariate analysis | 1998–2022 |
| Zhang et al. [26] | 2023 | China | Retrospective multicenter cohort | Invasive endocervical adenocarcinoma | 496* | 72 | 372 | 52 | 46.1 ± 10 (20–88) | 49.1 (95% CI 45.82–52.41) | 44.9 (95% CI 43.97–45.91) | FIGO I–III+ (complete staging available for 375 patients) | WHO 2020 histologic classification with HPV DNA detection by WTS-PCR and selected LCM-PCR, plus p16/PR immunohistochemistry review | Yes | Yes | No | HPV-independent adenocarcinoma according to WHO 2020 (gastric, clear cell, mesonephric) | HPV-associated adenocarcinoma according to WHO 2020 (usual, iSMILE, intestinal, signet-ring cell, mucinous NOS) | Gastric 39; clear cell 30; mesonephric 3 | Usual 257; iSMILE 57; mucinous NOS 51; intestinal 4; signet ring cell 3 | Pathology cohort (334 surgical resections; 162 punch biopsies); treatment not analyzed | NR | NR | NR | NR | NR | NR | NR | NR | NR | NR | NR | NR | NR | NR | NR | NR | NR | NR | No | No | NR | 2005–2010 |
| AIS, adenocarcinoma in situ; BMI, body mass index; CA125, carbohydrate antigen 125; CA199, carbohydrate antigen 19-9; CCI, Charlson comorbidity index; CCRT, concurrent chemoradiotherapy; CEA, carcinoembryonic antigen; CI, confidence interval; CR, complete response; CT, chemotherapy; CTx, chemotherapy; DFS, disease-free survival; DMFS, distant metastasis-free survival; DNA, deoxyribonucleic acid; DSS, disease-specific survival; ECA, endocervical adenocarcinoma; FIGO, International Federation of Gynecology and Obstetrics; HPV, human papillomavirus; HPVA, HPV-associated adenocarcinoma; HPVI, HPV-independent adenocarcinoma; HR, hazard ratio; IHC, immunohistochemistry; IECC, International Endocervical Adenocarcinoma Criteria and Classification; ISH, in situ hybridization; ISMC, invasive stratified mucin-producing carcinoma; iSMILE, invasive stratified mucin-producing intraepithelial lesion; LCM-PCR, laser capture microdissection polymerase chain reaction; LNM, lymph node metastasis; LRFS, local recurrence-free survival; LRRFS, locoregional recurrence-free survival; LVI, lymphovascular invasion; LVSI, lymphovascular space invasion; MRI, magnetic resonance imaging; mRNA, messenger ribonucleic acid; NAC, neoadjuvant chemotherapy; NDI, neighborhood deprivation index; NHPVA, non-HPV-associated adenocarcinoma; NOS, not otherwise specified; NR, not reported; OS, overall survival; PCR, polymerase chain reaction; PD, progressive disease; PFS, progression-free survival; PR, progesterone receptor; Rb, retinoblastoma protein; RFS, recurrence-free survival; RNA, ribonucleic acid; RRFS, regional recurrence-free survival; RT, radiotherapy; SD, stable disease; WHO, World Health Organization; WTS-PCR, whole tissue section polymerase chain reaction. * For selected studies, the total number preserves the full reclassified cohort including other, NOS, or unclassified cases when reported; the corresponding main table total may reflect the directly comparable analytic cohort only. ** Hazard ratios originally reported in the HPV-associated versus HPV-independent direction were converted to the HPV-independent versus HPV-associated direction by reciprocal transformation for consistency across studies. *** Local and distant failure data refer to the first site of failure only and should not be interpreted as cumulative recurrence counts. **** Lymph node status in this study refers to radiologic lymphadenopathy rather than pathologically confirmed lymph node metastasis. | | | | | | | | | | | | | | | | | | | | | | | | | | | | | | | | | | | | | | | | | | | |
